# Oral microbiome mediated inflammation, a potential inductor of vascular diseases: a comprehensive review

**DOI:** 10.3389/fcvm.2023.1250263

**Published:** 2023-08-30

**Authors:** Diego F. Gualtero, Gloria Inés Lafaurie, Diana Marcela Buitrago, Yormaris Castillo, Paula Katherine Vargas-Sanchez, Diana Marcela Castillo

**Affiliations:** Universidad El Bosque, Vicerrectoría de investigaciones, Facultad de Odontología, Unidad de Investigación Básica Oral-UIBO, Bogotá, Colombia

**Keywords:** microbiota, dysbiosis, bacteremia, *Porphyromonas gingivalis*, nitric oxide, cardiovascular diseases

## Abstract

The dysbiosis of the oral microbiome and vascular translocation of the periodontopathic microorganism to peripheral blood can cause local and systemic extra-oral inflammation. Microorganisms associated with the subgingival biofilm are readily translocated to the peripheral circulation, generating bacteremia and endotoxemia, increasing the inflammation in the vascular endothelium and resulting in endothelial dysfunction. This review aimed to demonstrate how the dysbiosis of the oral microbiome and the translocation of oral pathogen-induced inflammation to peripheral blood may be linked to cardiovascular diseases (CVDs). The dysbiosis of the oral microbiome can regulate blood pressure and activate endothelial dysfunction. Similarly, the passage of periodontal microorganisms into the peripheral circulation and their virulence factors have been associated with a vascular compartment with a great capacity to activate endothelial cells, monocytes, macrophages, and plaquettes and increase interleukin and chemokine secretion, as well as oxidative stress. This inflammatory process is related to atherosclerosis, hypertension, thrombosis, and stroke. Therefore, oral diseases could be involved in CVDs via inflammation. The preclinic and clinical evidence suggests that periodontal disease increases the proinflammatory markers associated with endothelial dysfunction. Likewise, the evidence from clinical studies of periodontal treatment in the long term evidenced the reduction of these markers and improved overall health in patients with CVDs.

## 1. Introduction

The oral microbiota fulfills essential homeostasis functions in the body. An increase in systemic nitrite can improve nitric oxide (NO) concentrations via oral microbiome by its capacity to reduce dietary nitrates; the NO is a vasodilatory solid and anti-inflammatory molecule that maintains vascular homeostasis (1). The dysbiosis of the oral microbiome with increased anaerobic Gram-negative microorganisms, such as *Porphyromonas gingivalis*, displaces commensal microorganisms with a high nitrate-reduction capacity (NRC) (2). However, *P. gingivalis* cannot reduce nitrate, which is significantly affected by the high concentration of nitrates (3). In addition, the dysbiosis of the subgingival microbiome induces the innate immune response, which impacts the vascular endothelium (4). Periodontitis is a multifactorial disease characterized by an inflammatory response that may lead to endothelial dysfunction (5, 6). The microbiome dysbiosis and the bacteremia and endotoxemia caused by periodontopathic microorganisms have been reported after tooth brushing and periodontal treatment in individuals with periodontitis (7, 8). These microorganisms can increase proinflammatory activation at a distance and are found in the arteries of patients suffering from periodontitis with atherosclerosis and a high risk of CVD (9–11). However, some concerns have arisen, such as what are these doing here? Do they cause or potentiate vascular lesions? How are they accomplishing this? This review discusses these and other topics to demonstrate the role of oral microorganisms and their potential to cause or worsen CVDs.

## 2. Periodontal disease: a chronic inflammatory disease

Periodontitis is a multifactorial chronic inflammatory mediated by a biofilm dysbiosis that generates diverse grades of tissue destruction (12). Severe periodontitis affects more than 700 million people worldwide, with a global prevalence of 10.8% (13). However, less severe forms of the disease affect 12%–55% of the population, with the prevalence varying by population (14). Periodontitis is among the most prevalent diseases worldwide with local and systemic consequences (15). Periodontitis is characterized clinically by migrating the junctional epithelium, allowing proliferation and cell migration on an altered connective tissue substrate, and inflammatory tissular status generating the depth of the gingival sulcus forming periodontal pockets. In periodontitis, the subgingival biofilm in the gingival sulcus causes tissue destruction, which expands to the periodontal ligament and alveolar bone, causing protein degradation and proteoglycan reduction (16). The 2018 world workshop diagnostic criteria are based on a multidimensional system including stages in ranges from 1 to 4 and depend on the severity of the disease and the complexity of its treatment and rehabilitation, considering clinical attachment loss (the connective tissue of the root cementum), percentage of bone loss, pocket depth and teeth lost due to periodontal disease as critical factors. It also has a grading system that provides information on the progression based on risk factors such as smoking, and diabetes used as modifiers for progression risk (17, 18).

### 2.1. Dysbiosis of oral biofilm in periodontitis and their effect on the cardiovascular system

The human oral cavity and gastrointestinal tract harbor the most abundant microbiota (19). The oral microbiome contains up to 750 species of microorganisms, including bacteria and other important microorganisms such as archaea, protozoa, fungi, and viruses. These can grow on the tongue, buccal mucosa, tonsils, and palate or hard surfaces such as teeth or dental prostheses. In a healthy environment, there is a balance between these species, with stable diversity and composition. However, this balance is disrupted in dysbiosis, resulting in a composition with an increase of commensals and a reduction in beneficial microorganisms (20).

It has been reported that the nitrites generated in the metabolism in the eubiotic microbiome contribute to systemic health, stimulating the circulatory system related to cardiometabolic health (21). This link occurs because hemoglobin sequentially oxidizes NO in the blood to NO2^−^ and NO3^−^. This NO3^−^ concentrated in the salivary glands is mixed with nitrate ingested from the diet and reduced to nitrite NO2^−^ by nitrate reductase enzymes oral bacteria. Salivary nitrite can be enzymatically reduced to NO, nitrous oxide (N_2_O), or dinitrogen (N_2_) in the mouth by denitrifying bacteria. Commensals such as *Actinobacteria*, including *Actinomyces* and *Rothia*; *Firmicutes*, such as *Veillonella* and *Streptococcus*; *Bacteroidetes*, such as *Prevotella* and some *Proteobacteria* that include the genus *Neisseria* and *Haemophilus* are the most critical microorganisms in eubiotic oral microbiome who improved the vasodilator function of the endothelium. The dysbiosis associated with periodontopathic microorganisms can move them and play an essential role in cardiovascular risk (21, 22).

**Figure 1:**
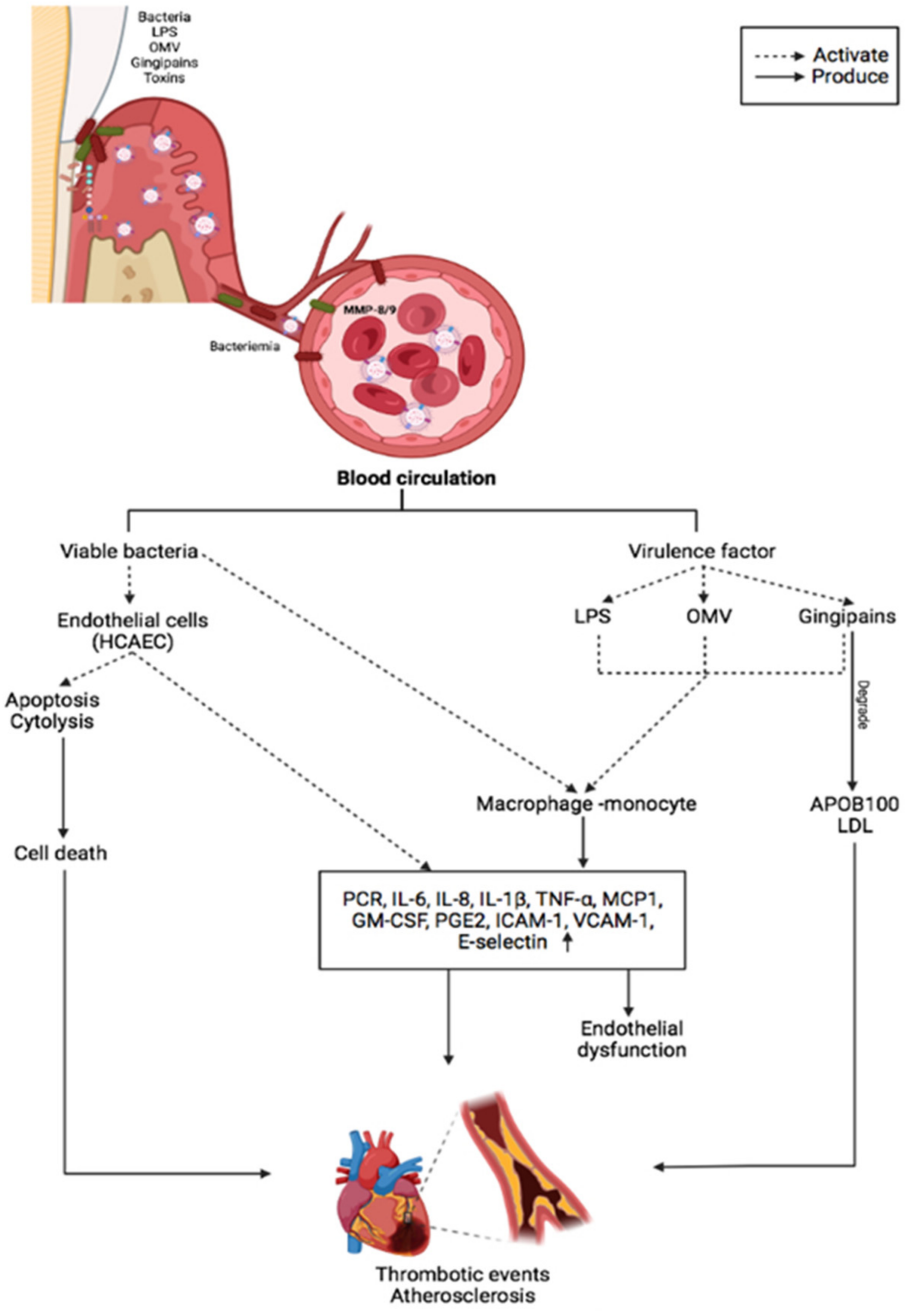
The main inflammatory effects of periodontopathogens and virulence factors on vascular endothelium and CVDs.

Pathobionts such as *P. gingivalis*, which cause dysbiosis and alter the bacterial community's relative abundances, can deregulate the inflammatory response (23–25). Subgingival microorganisms induce immune cell infiltration and inflammatory mediators in the periodontium (26, 27). Inflammatory markers can pass passively from the periodontal tissue to the peripheral circulation. Higher systemic circulating inflammatory burden of CVD risks such as interleukin (IL)-1β, IL-8, IL-6 and tumor necrosis factor-alpha (TNF-α) are directly associated with the periodontitis incipient lesions in adolescents and established periodontitis in adults (28, 29). Higher levels of circulating inflammatory markers have also been linked to patients with atherosclerosis with periodontitis (30). However, periodontal treatment reduces these biomarkers, particularly in those with CVD (31). These findings imply that periodontitis can elicit a systemic immune response that is not restricted to the localized lesion (32) (Figure 1).

*Porphyromonas* species are proteolytic and degrade proteins and peptides into amino acids, which are degraded to produce short-chain fatty acids (SCFA), ammonia, sulfur compounds, and indole (22). Although SCFAs are considered metabolites that preserve endothelial function, this appears to be differential. Butyrate decreased Nlrp3 inflammasome activation in mice's carotid arterial wall after consuming a Western diet, while acetate-like propionate markedly enhanced Nlrp3 inflammasome generated an activation and carotid neointimal formation in mice in the carotid arteries (33). Due to the high metabolism of SACS in the oral microbiome, this mechanism has been proposed as another mechanism associated with increased cardiovascular risk in patients with periodontitis.

### 2.2. Atopobiosis of periodontopathic microorganisms and their effect on the cardiovascular system

Dysbiosis transforms the microbiome's composition into an inflammatory state, whereas atopobiosis can induce inflammation by translocating microorganisms to locations other than their usual location (34). Gut and oral dysbiosis have been linked to the pathogenesis of several immune-mediated inflammatory diseases (35, 36). The microorganisms will likely enter the bloodstream from oral niches via mechanisms that allow translocation and atopobiosis (20). Chewing, brushing, periodontal probing, scaling and root planning, and dental extractions can transfer the bacteria to capillaries and other small blood vessels, allowing bacteria to enter the systemic circulation (20, 37). *P. gingivalis* is the most frequent microorganism during oral bacteremia, especially in patients with periodontitis (8, 38). It is the most common microorganism in the amniotic fluid, placental tissues (39–41), brains, cerebrospinal fluid (42, 43), and vascular tissues (44, 45).

Other different routes of entry of oral organisms have been proposed, such as pathway phagocytes or dendritic cells. Many oral organisms in many tissues are related to these pathways. Periodontal pathogens such as *P. gingivalis* and *F. nucleatum* can achieve intracellular survival and disseminate to distant sites (46). *P. gingivalis* can invade several cell lines, including human umbilical vein endothelial cells, KB cells, and the human oral epidermoid cell line (47). *P. gingivalis* invasion of endothelial cells and phagocytic cells within the atheroma is also critical in atherosclerosis progression in mice (46, 48). During the invasion in endothelial cells, microvillus-like extensions are observed around bacteria, followed by the engulfment within vacuoles, using fimbriae and *P. gingivalis* proteases, which are essential for invasion; *P. gingivalis* invasion is considered an evasion mechanism of the host immune response (49).

*P. gingivalis*, can also colonize different oral mucosa cells, including the tongue, floor of the mouth, and buccal mucosa (50). *P. gingivalis* strain ATCC 33277 activates pro-survival phosphatidylinositol-3-kinase (PI3K)/protein kinase B (AKT) pathway in the primary oral epithelial cells for its effective colonization; the microorganism regulates the inflammatory response promoting host cell survival through the PI3K/Akt pathway (51). *P. gingivalis* is associated with developing oral cancers by upregulating IL-8 and MMPs 1 and 2 levels and increasing metalloproteinase-9 stimulated by gingipains and by the induction of the PI3K/AKT signaling along with the epithelial-mesenchymal transition (EMT) (51). *F. nucleatum* also invades colorectal cancer cells (CRC) using galactose sugars, l-arginine, neutralizing membrane protein antibodies, or fap2 deletion and mobilizes immune cells, increasing IL-8 and CXCL1 production in tumors to generate their progression (52). Gal-GalNAc is overexpressed in CRC and recognized by Fap2 of *F. nucleatum*, identifies the tumors in a Fap2-dependent manner, and uses the hematogenous to reach the colorectal tumors (53).

During bacteremia, the innate response serves as the first line of defense (54). In healthy individuals, transient bacteremia of dental origin is common and is cleared rapidly and efficiently within a few minutes to an hour (55). Because of the blood speed and bacterial size, it is practically impossible for leukocytes to recognize, trap, and destroy bacteria in the bloodstream (56). Periodontal bacteria can sequester leukocytes and erythrocytes, which bind through complement 3b (C3b) via complement receptor type 1 and spread from the oral mucosa to the vascular tissues. The erythrocytes destroy the bacteria by oxidizing their membranes, and the Kupffer cells in the liver and the splenic lymphoid tissue macrophages engulf and digest them, resulting in their clearance (57). However, erythrocytes play a fundamental role in bacterial clearance due to their abundance. Therefore, they are the only cells capable of clearing the trillions of bacteria that can enter the blood during bacteremia in minutes (56). The complement system is another mechanism that aids in bacterial clearance and is an essential component of the innate immune response and is the first line of defense against bacterial infections. Despite its lack of specificity, complement selectively recognizes foreign pathogens and damaged cells damaged by using recognition molecules from the classical and alternate pathways and lectin receptors. Massive bacteremia has been observed in knockout mice lacking C5 and C3 due to their inability to induce phagocytosis or oxidative burst (58).

Recently, the atopobiosis of oral microorganisms was assessed by sequencing bacterial DNA and comparing it to subgingival biofilm samples and coronary balloons. Microbial diversity differed significantly between the two environments. However, the bacteria translocate between periodontal pockets and coronary arteries, and 17 phylotypes were identical between atheroma and subgingival samples (59). In vascular tissues, oral bacteria invade and activate endothelial cells and promote the transmigration of leukocytes that may harbor intracellular bacteria that induce the atheroma lesion formation (20). Another systemic mechanism of periodontitis is the presence of endotoxemia (60). The release of proinflammatory cytokines via lipopolysaccharide (LPS), LPS-binding protein (LBP), CD14, and toll-like receptor (TLR) activation (61). LPS-LBP is a complex that plays a crucial role in the innate immune response to bacterial challenges in the systemic and local environment by activating proinflammatory cytokines (62).

## 3. Periodontopathogens and CVD risk

Studies indicate that the adverse cardiovascular effects are due to a few putative or high-risk bacteria*: Aggregatibacter actinomycetemcomitans*, *Porphyromonas gingivalis*, *Tannerella forsythia*, *Treponema denticola or Fusobacterium nucleatum* (63)*. P. gingivalis*, and its virulence factors such as fimbriae (64, 65), LPS (66), gingipains (67), and outer membrane vesicles (OMVs) (68), *A. actinomycetemcomitans* with factors such as Leukotoxin (LtxA), cytolethal distending toxin (CDT), LPS and OMVs (69), and other microorganisms of dental biofilm as *F. nucleatum*, and *Eikenella corrodens*, and their virulence factors, migrate into the bloodstream and affect tissues and organs (7, 70, 71). Once in the systemic circulation, periodontal bacteria can be transported to distant sites, freely in the circulation or within circulating cells (36). Bacterial invasion can indirectly induce endothelial activation or generate endothelial dysfunction via systemic inflammation with increased acute-phase plasma proteins and proinflammatory cytokines. Furthermore, releasing bacterial products, such as OMVs, gingipains, or free soluble components, into circulation can induce proatherogenic responses in endothelial cells. Immune activation by the pathogen-derived GroEL heat shock protein (HSP) can also lead to an autoimmune response due to structural similarity between host HSP60 and GroEL (72, 73). *F*. *nucleatum* has been implicated in CVDs and is frequently detected in atherosclerotic plaques, as well as in a ruptured brain aneurysm (74). Genetic DNA from *P. gingivalis* and *F. nucleatum* in human aortic atherosclerotic lesions suggests a link between periodontal pathogens and CVDs (75).

OMVs also play a role in *P. gingivalis*-related systemic diseases. OMVs have been shown to activate Rho kinase in endothelial cells of umbilical veins via ERK1/2-and p38 mitogen-activated protein kinase, which may promote endothelial dysfunction and CVD (76). Preclinic experiments confirmed that *P. gingivalis* OMVs significantly increase vascular permeability. *P. gingivalis* can generate edema by altering the endothelial cell junctions such as platelet endothelial cell adhesion molecule-1 (77). The protease activity of *P. gingivalis* OMVs can cleave host proteins avoiding the immune response by degrading proinflammatory cytokines and disruption of the extracellular matrix, altering the tissue integrity, suggesting that these vesicles may play a vital role in the vascular damage (78). OMVs also promote vascular smooth muscle cell calcification via ERK1/2-RUNX2, inducing atherosclerosis (78, 79). *In vitro*, studies have demonstrated that OMVs from *P. gingivalis* can accelerate the foam cell formation in the murine macrophage and can induce the rupture of atherosclerotic plaque. *P. gingivalis* 381 also degrades fibrous caps and induce the matrix metalloproteinase (MMP)-9 activity in macrophages, which is involved in plaque disruption (80). *In vitro*, OMVs from *P. gingivalis* also induce platelet aggregation, which is essential in atherosclerotic plaque formation. In patients with periodontal disease, an increase in systemic inflammation, with elevated IL-1α and MMP-9 in plasma, causes a slight but significant decrease in cardiac function, dependent on MMP-9 (81). The proteolytic and oxidative activity of *P. gingivalis* also increased protein oxidation forming two apoB-100 N-terminal fragments that modified the LDL, which induce cell proliferation and could influence atherosclerosis (82). Likewise, the degradation of apoB-100 by Rgps gingipain plays a crucial role in promoting atherosclerosis by *P. gingivalis* infection (83) (Figure 1).

## 4. Endothelial dysfunction mediated by periodontopathic microorganisms

The vascular endothelium comprises monolayer endothelial cells that rest the intima formed by a matrix of proteoglycan. Endothelial cells perform various functions, including vascular tone, permeability, catabolic metabolism, SMC proliferation, angiogenesis, inflammation, white cell trafficking, fibrinolysis, thrombosis, and platelet activation (84). Therefore, the endothelial injury could alter their functionality, causing atherosclerosis (85). Furthermore, endothelial dysfunction can be caused by several factors, such as hyperlipidemia, oxidative stress, and inflammation due to bacteremia and endotoxemia (86).

Both periodontitis and CVDs are chronic inflammatory diseases. Biochemical and physiological analyses, including *in vitro* experiments, animal models, and clinical studies, establish a significant impact of periodontal pathogens, their virulence factors, and bacterial endotoxins on CVD mechanisms such as systemic inflammation, oxidative stress, endothelial dysfunction, foam cell formation, lipid accumulation, atherothrombosis, and vascular remodeling (6, 87). However, there is insufficient evidence to conclude that periodontitis causes CVDs. In this way, some studies have shown that periodontitis contributes to CVD through systemic bacterial exposure. For instance, the meta-analysis by Mustapha et al. demonstrated that elevated markers of systemic bacterial exposure, such as IgG to *P. gingivalis*, are associated with coronary heart diseases in periodontal patients (88).

In a population-based cohort study, Holtfreter et al. revealed a significant association between pocket probing depth and flow-mediated dilation of the brachial artery (6). Moreover, periodontal therapy has been shown to reduce inflammatory markers in CVDs. A previous meta-analysis demonstrated a significant weighted mean difference in CRP, proinflammatory cytokines, fibrinogen, total cholesterol, high-density lipoprotein-cholesterol, and improved endothelial function after periodontal therapy (32). In this context, we can speculate that periodontal pathogens promote endothelial dysfunction mechanisms to increase CVD risk.

Lipopolysaccharides (LPS) are biomolecules found in the outer membranes of anaerobic Gram-negative microorganisms composed of a lipidic (Lipid A) and a polysaccharide (O-antigen) region. Several studies have shown that Lipid A has heterogeneous structures; for example, some bacteria, such as *Escherichia coli*, have Hexa-acylated lipid A, whereas oral bacteria, such as *P. gingivalis*, have Penta- and Tetra-acylated lipid A (89, 90). The various structures of Lipid A from LPS are associated with multiple cell recognition and innate immune responses.

LPSs bind to TLRs in host cells, and several studies have shown that the classic structure of Hexa-acylated lipid A matches TLR-4, whereas Penta- and Tetra-acylated lipid A matches TLR-2. There are controversial studies on LPSs from *P. gingivalis* (LPS-*Pg*) and their TLRs; some studies show that LPS-*Pg* activates cells via TLR2, whereas others show that it activates cells via TLR4. Nonetheless, LPS-*Pg* heterogeneity enables recognition via Penta-acylated lipid A with TLR4 and Tetra-acylated lipid A with TLR2. LPSs from atherosclerosis-associated bacteria inhibit TLR4 and active endothelial cells via TLR2, resulting in a proinflammatory atherosclerotic response mediated by chemokines and cytokines (89, 90). Endothelial secretion of IL-8, monocyte chemoattractant protein-1 (MCP-1), IL-6, and TNF-α aids in leukocyte tracking and adhesion.

In bidimensional and three-dimensional models, human coronary artery endothelial cells (HCAECs) have low inflammatory responses to LPS-*Pg* (91). Nonetheless, when HCAECs were exposed to multiple doses of *P. gingivalis*, they produced significant amounts of MCP-1, IL-6and granulocyte-macrophage colony-stimulating factor (92). Other microorganisms, such as *A. actinomycetemcomitans* and *E. corrodens*, have a greater proinflammatory capacity than *P. gingivalis*. Treating HCAECs with *A. actinomycetemcomitans* and *A.a*-LPS IL-8 and IL-6 secretion increased via nuclear factor kappa beta-p65 (NF-κB p65) activation. However, this inflammatory response was inhibited by rosuvastatin due to the atheroprotective factor Kruppel-like factor 2 (93). A recent study demonstrated that *A. actinomycetemcomitans* infection increased transforming growth factor beta 1, IL-8, MCP-1, and IL-6 secretion in HCAECs in a dose-dependent manner in a 3D model. This proinflammatory response stimulated the THP-1 monocyte activation, adhesion, and migration (94). Similarly, *E. corrodens*-LPS activates HCAECs pathway TLR4, ERK, and NF-kB p65, inducing a pro-atherosclerotic endothelial response and monocyte adhesion (95). Previous evidence suggests that inflammation induced by oral pathogens and microorganisms may promote endothelial dysfunction and CVD progression (Table 1). A plausible mechanism for periodontopathogens' ability to induce endothelial dysfunction associated with CVDs and stroke is depicted in Figure 1.

**Table 1:** Periodontopathogens factors related to endothelial dysfunction.

Factors	Target	Mechanisms	Outcome	References
Dysbiosis of oral biofilm	Endothelial cells	Alteration in the reduction of nitrites of the diet.	Hypertension	(21)
Dysbiosis of oral biofilm	Endothelial cells	Proinflammatory cytokines.	Endothelial dysfunction and atherosclerosis	(23–32)
Dysbiosis of oral biofilm	Endothelial cells	Nrlp3 inflammasome activation by SCFAs.	Endothelial dysfunction	(121)
Bacteremia by *P. gingivalis*	Leukocytes and erythrocytes receptors	Sequestration and internalization of bacteria by complement receptor.	Invasion of the vascular tissues	(57)
Bacteremia by oral pathogens	Endothelial cells	Expression of cell adhesion molecules and chemokines, promoting the transmigration of leukocytes	Invasion of the vascular tissue	(20)
Bacteremia by oral pathogens	Platelets and erythrocytes	Activation and aggregation of cells.	Clot formation and pro coagulation	(103, 104)
*P. gingivalis*, *F. nucleatum* *A. actinomycetemcomitans* *Streptococcus sanguinis*	Platelets activation	Direct binding to platelet receptors, indirect binding to fibrinogen, fibronectin, and von Willebrand factors of LPS, gingipains, and OMVs.	Procoagulation	(106, 107)
Bacteremia *P. gingivalis, A. actinomycetemcomitans*	Endothelium, foam cell	Stress oxidative, ox-LDL, ROS.	Formation of atherosclerotic plaque	(119–121)
Endotoxemia by LPS	Endothelial cells and monocytes.	Activation via CD14.	Secretion of pro-inflammatory cytokines, adhesion, and migration monocyte	(62, 91–95, 122)
OMVs and gingipains	Endothelial cell	Cleave of endothelial adhesion molecules as a PECAM-1 (CD31) and VE-cadherin (CD144). Suppressed eNOS expression by Activation of ERK1/2 and p38 MAPK.	Increased endothelium permeability in vitro	(77, 78)

## 5. Macrophage–monocyte activation by periodontopathogens and cardiovascular risk

Macrophages play an active role as effectors and regulators in different phases of inflammation. Together with other cells, they are phagocytic cells that effectively modulate innate and adaptive immune responses, promoting inflammatory resolution and tissue healing (96). In periodontitis, macrophages secrete chemotactic and different cytokines such as RANTES (Regulated upon Activation, Normal T Cell Expressed and Presumably Secreted), MCP-1, macrophage inflammatory protein (MIP)-1α, MIP-3α, MIP-1β, and IL-8, which activate and migrate leukocytes, and proinflammatory cytokines such as IL-1β, TNF-α, and IL-6, which activate osteoclastogenesis processes and the degradation of periodontal tissue (97).

OMVs are essential virulence factors secreted into the external environment and serve as a generalized secretion and transport system for other virulence factors such as fimbriae, endotoxin as LPS, and enzymes, including gingipains. The role of this bacterium and its vesicles in the host immune response and tissue destruction by *P. gingivalis* infection has been well investigated. They have been shown in an *in vitro* model using the human macrophage cell line U937 the induction of proinflammatory cytokines associated with osteoclastogenesis, alveolar bone resorption, and tissue destruction (98, 99). In atherosclerosis-related endothelial dysfunction, monocytes migrate through the endothelium, differentiate into macrophages in the subendothelial tissue, and then convert to foam cells. LPSs from periodontal pathogenic bacteria induce acceleration in foam cell formation (100). LPSs from *A. actinomycetemcomitans* have been shown to synergistically interact.

LPSs play a role in developing atherosclerotic and CVD, including coronary artery disease, cerebrovascular disease, peripheral artery disease, and aortic atherosclerosis, increase TLR expression and respond to TLR agonists inducing significant inflammatory lesions (101). These roles and their plasticity make macrophages attractive targets for preventing and stabilizing existing atherosclerosis (102).

## 6. Prothrombotic state induced by periodontopathogens

Platelets play a role in various phases of the atherosclerotic process and vascular thrombosis leading to myocardial infarction. In periodontitis, low-grade systemic inflammation contributes to platelet activation, aggregation, and a procoagulant state (103, 104). Platelet activation is associated with the decrease in the release or inactivation of nitric oxide, as well as the release of platelet agonists. Platelet-derived substances, such as nitric oxide, cytokines, growth factors, chemokines, metalloproteinases, histamine, and selectins, actively participate in immune and inflammatory reactions (105, 106).

Epidemiological studies in patients with chronic periodontitis have revealed an increase in the mean platelet volume and platelet count, which has been linked to the production of cytokines such as IL-3 or IL-6, which in turn regulates megakaryocyte ploidy, producing more giant and reactive platelets (104, 105). Using *in vitro* studies, animal models, and clinical studies, *P. gingivalis*, *F. nucleatum*, and *A. actinomycetemcomitans* have been identified as the most commonly associated periodontopathogens with platelet activation and aggregation. The following mechanisms of pathogen–platelet interaction have been described: (1) direct bacterial binding to one of the platelet receptors; (2) indirect binding via other mediators such as fibrinogen, fibronectin, and von Willebrand factor (vWF); and (3) binding of bacterial products and toxins such as LPS, gingipains, and OMVs (80, 106–108).

Pathogens have been shown to express surface proteins, allowing them to bind directly to platelets. In this regard, *P. gingivalis* produces kazal-type (KSPI) serine proteasa and Arg-gingipains, RgpA, and RgpB, which, when bound to hemagglutinin/adhesion, stimulate platelets via Par-1 and Par-2, producing inositol trisphosphate and intracellular calcium downstream (107). Moreover, Naito et al. demonstrated that this microorganism could express the adhesin protein Hgp44, which can activate platelets by binding to glycoprotein (GP) Ib–IX–V, a vital platelet receptor essential in primary hemostasis due to its high affinity for vWF or the FcγRIIa receptor, a potent activator of hemostasis, followed by activated platelet elimination (108). Furthermore, *S. sanguinis* expresses highly glycosylated serine-rich protein A, which can bind to GPIbα (109), or indirectly induce platelet aggregation via the complement pathway. This interaction can aid in bacterial destruction, but it can also activate platelets inducing procoagulant factors such as the prothrombinase complex on the cell surface generating platelet–bacteria binding by complement molecules inducing an immunological mechanism rather than a hemostatic mechanism, highlighting platelets' dual role (110).

On the other hand, platelets can modulate the responses of other cells such as leukocytes and endothelial cells, via TLR2 and the GP ligand P-selectin/P-selectin (111). Platelets express TLR1, TLR2, TLR4, TLR6, TLR8, and TLR9, and the interaction of TLR2 and TLR4 with LPS from *A. actinomycetemcomitans* and *P. gingivalis* causes platelets to release cytokines such, as IL-1, TNF-α, and soluble CD40 ligand (sCD40l) (111, 112). Plasma sCD40l can predict recurrent cardiovascular events such as myocardial infarction and stroke, and arginine gingipains (RgpA and RgpB) of *P. gingivalis* can cause platelet activation and aggregation. Arg-ingipains enter the circulation and increase intracellular calcium levels in platelets by activating the protease-activated receptor-1 (PAR-1) and PAR-4 receptors, activate prothrombin, factor X and protein C, promoting thrombosis via thrombin release (113, 114). The vascular responses, upregulates endothelial cell adhesion molecule expression, and increases IL-1β, TNF-α, and thromboxane secretion, resulting in macrophage recruitment, aggregation, and platelet adhesion (114). Sharma et al. demonstrated that OMVs activate platelet aggregation. In contrast, fimbria is essential in bacterial adhesion to platelets via fibrinogen bridges that bind to the integrin receptor on the platelet surface. Most likely, this is what makes OMVs interact with platelet membrane receptors, which cause platelets to be stimulated and release dense and alpha granules and aggregate (80).

Platelets also link thrombosis and inflammation by producing platelet microparticles (PMPs). PMPs can release immunomodulatory factors such as RANTES, IL-1β, and CD40l. They can also modulate the activation of inflammatory cells such as neutrophils. In addition, bacterial infection with *P. gingivalis* induces PMP formation (112), so more studies are required to fully understand their role and association with periodontal pathogens. Studies have demonstrated that bacteria-induced platelet aggregation differs from that induced by conventional agonists. The above is recommended as a “binary” aggregation, which means that no aggregation is observed below a specific bacterial density, and aggregation is already maximal above that density (105). Therefore, it remains controversial. Several studies demonstrate that bacterial stimulation may not result in aggregation but rather in a more specific inflammatory response by activating the platelet TLR pathway (105).

We evaluated the effect of live*-P. gingivalis W83* and live-*A. Actinomycetemcomitans* ATCC 29522 (10^6^ CFU/kg) in an intraperitoneal infection model for six weeks in Wistar rats (ethical act No. 657), on platelet aggregation in platelet-rich plasma (PRP) using the spectrophotometric technique of aggregometry (115), against agonists adenosine diphosphate (ADP) (10 µM), collagen (10 µg/ml), arachidonic acid (AA) (150 µg/ml) and U46619 (2.10 µM thromboxane analog). The results demonstrate that after six weeks of treatment at the cumulative induction dose of live*-P. gingivalis* W83 and live*-A. Actinomycetemcomitans* on platelet aggregation in Wistar rats significantly increases platelet aggregation against agonists AA and U46619. In turn, it was shown that both periodontopathogens inhibit platelet aggregation in the presence of collagen (Table 2), which may be related to the “binary” effect of aggregation already described (105).

**Table 2:** Effect on platelet aggregation.

Treatment	ADP (%)	Collagen (%)	AA (%)	U46619 (%)
Control	97.5	92.5	98.5	88.5
1	82.5	44*	129.5*	126.3*
2	78.3	58.5*	131.5*	136*

Effect on platelet aggregation induced by ADP (10 µM), collagen (10 µg/ml), AA (150 µg/ml), and U46619 (10 µM) in PRP from Wistar rats treated at cumulative doses for 6 weeks with 1. *live-P. gingivalis*, 2. *live-A. Actinomycetemcomitans*. Each point represents the average ± SE of *n* = 5; *p < 0.05 with respect to the control (uninfected rats).

## 7. Endothelial oxidative stress

Oxidative stress and chronic inflammation are significant contributors to atherosclerosis. The oxidation of polyunsaturated fatty acids (PUFAs) by free radical lipid peroxidation (LPO) in lipoproteins to phospholipids and cholesterol esters forms a fatty streak in atherogenesis. LDL-containing PUFAs are prime targets for reactive oxygen species (ROS), resulting in non-enzymatic oxidation processes known as LPO (116–118). LPO can be induced by multiple endogenous factors such as lipoxygenases, cyclooxygenases, myeloperoxidases, NADPH oxidases, and ROS generated by the mitochondrial electron transport chain (118).

Vascular oxidative stress and inflammation can be induced by oral microorganisms such as *P. gingivalis* and *A. actinomycetemcomitans* (119). *A. actinomycetemcomitans* was used to assess LPO production in the mouse aorta (73). The increase in oxidized LDL (ox-LDL) levels, with an increase in NADPH expression or oxidative stress in the aorta of Aa-infected mice, suggests that *A. actinomycetemcomitans* plays a significant role in oxidative stress and LDL oxidation. In spontaneously hyperlipidemic mice with apolipoprotein E deficiency exposed to LPS and *A. actinomycetemcomitans*, ROS production depends on NOX (nicotinamide adenine dinucleotide phosphate oxidase) and myeloperoxidase activities, accelerating the formation of atherogenic plaques (118).

In *P. gingivalis*, ROS generation is dependent on the interaction of bacterial gingipain R with platelets and platelets with neutrophils, which promotes foam cell formation by increasing ox-LDL absorption (73). In addition, it contributes to the progression of atherosclerotic plaques by stimulating MCP-1 release in vascular endothelial cells via NOX-mediated superoxide anion production, followed by activation of multiple signaling pathways such as p38, c-Jun N-terminal kinase, and NF-κB (118). Recent studies have shown that *P. gingivalis* OMVs are systemic proinflammatory and prooxidant effectors of endothelial responses and that they upregulate proinflammatory cytokines such as TNF-α and IL-6. Their secretion has also been linked to uncontrolled oxidative stress via eNOS downregulation and iNOS upregulation, implying an uncoupling of NO synthase (120).

## 8. Association of periodontitis with CVDs

CVDs are the leading cause of death globally (121). CVDs are pathological disorders that damage the heart and blood vessels, including coronary heart disease, cerebrovascular disease, and peripheral artery disease associated with atherosclerosis (122). In 1989, Mattila et al. were the first to identify oral infection, especially periodontal disease, as an independent predictor of myocardial infarction risk. Since then, several observational studies on the association between periodontal disease and CVDs have been published (123). Thus far, several systematic reviews have demonstrated that periodontal disease has a minor association with CVDs, with odds ratios (ORs) ranging from 1.14 to 1.34 (124, 125). However, subgroup analyses revealed that exposure to infection for more than 15 years was associated with an increased OR of 1.67 (95% confidence interval (CI): 1.27–2.2) (124). Moreover, systemic bacterial exposure based on IgG antibodies against periodontal pathogens raises the risk to 1.75 (95% CI: 1.32–2.34) (88). When periodontitis or edentulism was used as a mortality indicator, there was an increased risk of all-cause mortality and CVD mortality. However, a higher risk of heart disease (RR: 2.58, 95% CI: 2.20–.03) and cerebrovascular diseases (RR: 3.11, 95% CI: 2.42–3.98) were detected (126). Likewise, patients with periodontitis and erectile dysfunction presented a higher number of cardiovascular adverse events adjusted by age and previous cardiovascular disease (127).

Due to the difficulty of conducting clinical trials to assess the impact of periodontal treatment, only a multicenter clinical trial known as the Periodontitis and Vascular Events (PAVE) was conducted as a pilot study (128). Although no differences in the number of cardiovascular events were observed between periodontally treated patients and control over 24 months, periodontal care exhibited a significant reduction in the percentage of individuals with elevated high-sensitivity CRP (hs-CRP) over 3 mg/L at 6 months (129). However, a larger sample size and a longer time frame are required to assess the effect of periodontal treatment on new cardiovascular events.

The relationship between inflammation and endothelial dysfunction has been widely demonstrated (130, 131). Several clinical studies later confirmed this hypothesis, revealing a critical epidemiological association between inflammatory markers such as CRP and acute ischemic events (132–134). CRP has been used as a universal marker to predict the risk of coronary heart disease in intermediate-risk individuals (135). The effects of hs-CRP on vascular risk are linear, with higher values above 3 mg/L predicting worse cardiovascular outcomes (135). Moreover, reducing this marker with anti-inflammatory drugs such as statins reduced the risk of coronary events in patients with high CRP (>2 mg/L) but normal cholesterol levels (136). CRP is produced in the liver in response to inflammatory molecules, especially IL-6, activated mainly by innate immune responses to infectious agents or secondary to systemic inflammatory diseases (135–137). However, hs-CRP is produced in the SMCs of the coronary artery due to endothelial dysfunction (138). Because CRP is not produced in the periodontal tissue, hs-CRP detected in the crevicular fluid is most likely of systemic origin (139). However, in patients with periodontitis, local proinflammatory cytokines and transient bacteremia overexpression may be associated with increased oxidative stress and CRP levels (140). Recently, Machado et al. showed that chronic and aggressive periodontitis is consistently associated with higher hs-CRP levels. Patients with aggressive periodontitis had hs-CRP levels more than 50% higher than patients with chronic periodontitis. Intensive nonsurgical periodontal treatment increased hs-CRP levels immediately, followed by a decrease after treatment. Non-intensive treatment reduced hs-CRP for up to 180 days. These findings indicate that periodontitis is associated with systemic inflammation using the serum hs-CRP levels as a marker (141).

**Figure 2:**
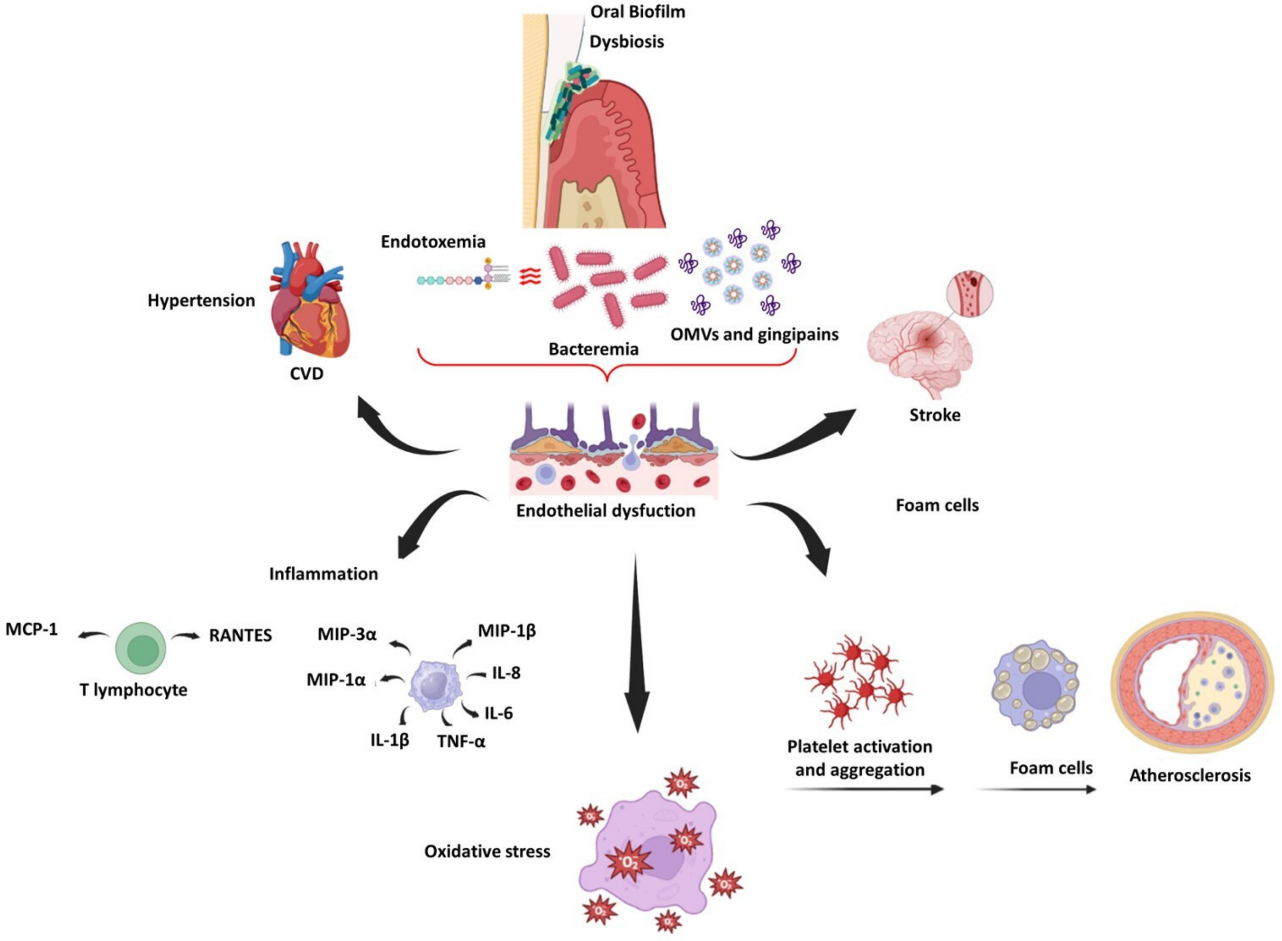
Schematic representation of endothelial dysfunction related to oral dysbiosis, CVDs, and stroke.

Periodontitis also induces endothelial dysfunction in rats, as evidenced by reduced eNOS expression and increased iNOS and COX-2 expression (142, 143). In humans, Tonetti et al. compared patients with severe periodontitis who received intensive periodontal treatment (IPT) to a control group who received community-based treatment. Endothelial function was assessed using flow-mediated dilatation (FMD), and inflammatory, coagulation, and endothelial activation markers were assessed before and after treatment. FMD was higher in the IPT group than in the control group by the second month. There was a significant correlation between the change in FMD and improved periodontal status. However, few changes were observed in inflammatory markers 6 months after therapy (144). After 6 months of intervention, the effects of periodontal treatment on endothelial function in patients with a recent ST-segment elevation myocardial infarction were observed in individuals with severe periodontitis (145). Similarly, periodontal therapy improved endothelial function in patients with a recent myocardial infarction without causing adverse clinical events (146). Figure 2 illustrates the main inflammatory effects of periodontopathogens and virulence factors on vascular endothelium and CVDs.

## 9. Association between periodontal disease and hypertension

Another effect of periodontal disease in CVDs is arterial hypertension. Periodontitis causes inflammation and oxidative stress, which causes vascular changes such as arterial stiffness, vascular dysfunction, and hypertension (147, 148). Epidemiological observational studies have supported the association between periodontal disease and hypertension (149, 150). Moreover, IPT significantly reduced the systolic blood pressure (SBP) in patients with the periodontal disease after treatment compared to those who did not receive treatment, which correlated with periodontal status improvement. Periodontal treatment also improved diastolic pressure and endothelial function (FMD), with lower levels of circulating interferon-gamma and IL-6, as well as activated and immunosenescent CD8^+^ T-cells, both of which have been linked to hypertension (151, 152). In 2021, an integrative study of evidence based on eight randomized controlled trials found that IPT improved SBP and diastolic blood pressure in individuals with hypertension and prehypertension, with significant reductions in CRP and improvement in endothelial function after periodontal treatment. This evidence supports the role of periodontal treatment in improving endothelial dysfunction in patients with CVDs (149).

In a multiethnic cohort study on the direct assessment of subgingival periodontal bacterial load and its relationship to BP, which restricted clinical periodontal assessment bias, a strong positive association was demonstrated between increased subgingival colonization by, *P. gingivalis*, *T. forsythia*, *T. denticola,* and *A. actinomycetemcomitans* and arterial hypertension (148). To our knowledge, this was the first study to link bacterial load to increased blood pressure. Periodontitis is associated with systemic inflammation, the mediators can affect endothelial function (92). Clinical evidence suggests that periodontitis affects systemic endothelial function, which may affect hypertension, with some reports suggesting possible direct effects of bacteremia-related *P. gingivalis* in mediating vascular dysfunction and immune response, resulting in elevated BP, vascular inflammation, and endothelial dysfunction (147).

As discussed above, an imbalance in NO bioavailability associated with oral microbiome dysbiosis is associated with some cardiovascular and metabolic diseases (21, 22). A representative population study using data from the third National Health and Nutrition Examination Survey (NHANES III) demonstrate a significant association of elevated BP with antibodies against *Campylobacter rectus*, *Veillonella parvula*, and *Prevotella melaninogenica*. *C. rectus* resulted in the strongest association with BP (153). In another study, in periodontitis patients, high BP was associated with higher *P. intermedia*, *P. gingivalis*, and *F. nucleatum* counts (154). This dysbiosis can displace the denitrifying bacteria, reduce the NO, and alter arterial vasodilatation (21). Different publications have been realized to establish the impact of mouthwashes on the elevation of BP; The use of chlorhexidine, even at lower concentrations, inhibited the nitrate activity and the *Veillonella dispar* counts, however, the activity of salivary nitrite production was not affected (155). Other mouthwashes, such as essential oil, povidone-iodine, and cetylpyridinium chloride, have little effect on nitrate-reducing activity (156). The use of chlorhexidine mouthwash for one-week generated changes in the microbiome of the tongue and changes in systolic pressure in normotensive individuals. Otherwise, the individuals who cleaned the tongue regularly resulted in an enrichment of nitrate-reducing bacteria on the tongue (157). However, the tongue clean may disrupt the papillary surface and favor chlorhexidine penetration, resulting in a significant alteration of bacterial community and greater SBP (157).

Regarding the local renin–angiotensin system in gingival tissue, there may be another pathogenic link between the two conditions under investigation (147), in addition to a possible variable risk of periodontitis among different renin–angiotensin system genetic polymorphisms. Moreover, Viafara et al. demonstrated that repeated exposure to live *P. gingivalis* or LPSs induces the release of proinflammatory cytokines and angiotensin II in HCAECs and mediators of systemic inflammation such as CRP, IL-6, and TNF-α, contributing both to endothelial dysfunction (92). This could be linked to the Th1 response induced by bacterial antigens such as *P. gingivalis* by increasing sensitivity to the suppressive pro-hypertensive insult evoked by low doses of angiotensin II (147). Therefore, it supports the “two-hit” hypothesis, which states that immune activation at sites of chronic inflammation exacerbates responses to low-dose angiotensin II, establishing a link between chronic immune activation and hypertension.

## 10. Conclusions

Periodontitis and CVDs are both inflammatory diseases caused by the systemic circulation of periodontopathogens and their virulent factors, which can cause endothelial dysfunction via ILs, cytokines, oxidative stress, monocyte and macrophage activation, plaque aggregation, and cellular proliferation. These events could be related to CVDs such as atherosclerosis and hypertension. Periodontal treatment reduces inflammatory markers related to CVDs and may reduce the risk of CVD events. Dysbiosis of the oral microbiome in saliva and tongue in periodontal patients plays a vital role in the loss of balance that regulates blood pressure mediated by NO, which induces endothelial dysfunction. A controlled preventive therapy on other cardiovascular markers could significantly contribute to managing cardiovascular patients with periodontitis. This effect should be demonstrated in the future.
